# Artificial Light for Improving Tomato Recovery Following Grafting: Transcriptome and Physiological Analyses

**DOI:** 10.3390/ijms242115928

**Published:** 2023-11-03

**Authors:** Xiaotao Ding, Chen Miao, Rongguang Li, Lizhong He, Hongmei Zhang, Haijun Jin, Jiawei Cui, Hong Wang, Yongxue Zhang, Panling Lu, Jun Zou, Jizhu Yu, Yuping Jiang, Qiang Zhou

**Affiliations:** 1 Shanghai Key Laboratory of Protected Horticultural Technology, Horticultural Research Institute, Shanghai Academy of Agricultural Sciences, Shanghai 201403, China; dingxiaotao@saas.sh.cn (X.D.); miaochen@saas.sh.cn (C.M.); 13851997535@163.com (L.H.); zhanghongmei@saas.sh.cn (H.Z.); jinhaijun@saas.sh.cn (H.J.); cuijiawei@saas.sh.cn (J.C.); wh_yxl1201@126.com (H.W.); xuezylemon@foxmail.com (Y.Z.); lpl2245@163.com (P.L.); yy2@saas.sh.cn (J.Y.); 2 College of Ecological Technology and Engineering, Shanghai Institute of Technology, Shanghai 201418, China; lrg19971118@163.com; 3 College of Sciences, Shanghai Institute of Technology, Shanghai 201418, China; zoujun@sit.edu.cn

**Keywords:** healthy index, graft, plant hormones, plant morphogenesis, gene expression, light

## Abstract

Grafting is widely used to enhance the phenotypic traits of tomatoes, alleviate biotic and abiotic stresses, and control soil-borne diseases of the scion in greenhouse production. There are many factors that affect the healing and acclimatization stages of seedlings after grafting. However, the role of light has rarely been studied. In this study, we compared the effects of artificial light and traditional shading (under shaded plastic-covered tunnels) on the recovery of grafted tomato seedlings. The results show that the grafted tomato seedlings recovered using artificial light had a higher healthy index, leaf chlorophyll content, shoot dry weight, and net photosynthetic rate (P_n_) and water use efficiency (WUE) compared with grafted seedling recovered using the traditional shading method. Transcriptome analysis showed that the differentially expressed genes (DEGs) of grafted seedlings restored using artificial light were mainly enriched in the pathways corresponding to plant hormone signal transduction. In addition, we measured the endogenous hormone content of grafted tomato seedlings. The results show that the contents of salicylic acid (SA) and kinetin (Kin) were significantly increased, and the contents of indoleacetic acid (IAA) and jasmonic acid (JA) were decreased in artificial-light-restored grafted tomato seedlings compared with those under shading treatments. Therefore, we suggest that artificial light affects the morphogenesis and photosynthetic efficiency of grafted tomato seedlings, and it can improve the performance of tomato seedlings during grafting recovery by regulating endogenous hormone levels.

## 1. Introduction

Grafting is an ancient technique consisting of the union of a plant shoot (scion) and a root system (rootstock) [1]. It is a possible solution for mitigating the response of horticultural crops relative to environmental factors, producing significant genetic/metabolic effects that alter the bioagronomic response of many crops essential to human nutrition [2,3].

The tomato (*Solanum lycopersicum* L.) is the world’s second most produced vegetable and the first in economic trade exchanges. As reported by FAOSTAT, the total tomato production in China (2021) amounted to 67,636.72 million kilos from 1.14 million cultivated hectares (https://www.fao.org/faostat/en/#data/QCL, accessed on 17 July 2023), accounting for 26.34% of the world’s tomato production and 18.14% of the world’s harvest area. Tomato grafting is a useful and popular method for improving plant performance in greenhouse production. It is largely used for inducing the beneficial phenotypical traits of the rootstock to the scion, such as its big root size, improved yield, fruit quality, and resistance to biotic and abiotic stresses [4].

High-quality grafted seedlings can ensure that the advantages of grafting are used in tomato production. The successful and fast connection between the rootstock and scion, a process that takes place during the healing and acclimatization period, was very important for obtaining strong grafted seedlings [5]. Optimizing climate factors during recovery (the healing and acclimatization stages after grafting) is vital for healthy seedling production [6]. Normally, specific environmental conditions such as high humidity, low radiation, and suitable temperature are required to prevent excessive water loss and wilting of the scion. Thus, healing and acclimatization are generally carried out under shaded plastic-covered tunnels [7]. However, the accuracy of climate control (such as light, temperature, humidity, and radiation) is poor for this system. At the same time, light is the most obvious diurnal and seasonal variation [8]. The regulation of light intensity during the growth and vitality of grafted seedlings is also currently very neglected [9].

Light plays a crucial role in the growth and development of plants, providing both energy and signal [10,11]. Light intensity is one of the important factors that affects plant growth and quality, including morphology, photosynthetic characteristics, and nutrient content [12,13]. Many weak grafted seedlings were identified under shaded plastic-covered tunnels during cultivation practices. Therefore, replacing the use of traditional shaded plastic-covered tunnels and creating new tomato grafting methods under which the recovery of seedlings at the healing and acclimatization stages is extremely urgent for improving the quality of grafted tomato seedlings.

Previous studies have evaluated the influence of light on growth during healing and acclimatization and the morphogenesis of grafted pepper [5], watermelon [6,7], and eggplant [14], mainly focusing on light quality. Contrastingly, the potential regulatory mechanisms underlying light that relate to transcriptome sequencing and plant hormones for plants in recovery, especially for grafted tomato seedlings, have been studied infrequently. 

In this study, we analyzed the effects of artificial light and traditional shading methods on the survival, growth morphology, photosynthesis, and chlorophyll fluorescence of tomato seedlings during seedling grafting and healing. We then performed transcriptome analysis to characterize the grafted tomato seedlings under artificial light, which showed high healing and adaptability rates, and compared it to the transcriptome of grafted seedlings under traditional shading. Furthermore, endogenous hormones were measured in grafted tomato seedlings under artificial light and traditional shading. We found that the grafted tomato seedlings under artificial light were mediated via the activation of the genes in hormone signaling pathways and the regulation of endogenous hormone content during healing and adaptation. These results enhance our comprehension of the potential benefits of artificial light on the healing and acclimation of grafted tomato seedlings.

## 2. Results

### 2.1. Influence of Different Recovery Methods on Grafted Tomato Seedling Growth Parameters

**Figure 1:**
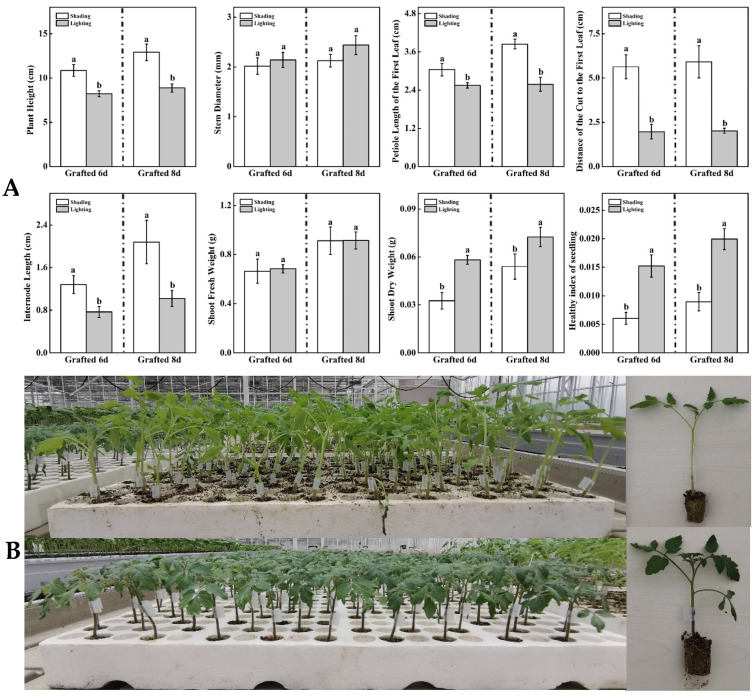
Effect of different recovery methods on the growth parameters and phenotype of grafted tomato seedlings. (**A**): Shading represents tomato seedlings recovered in a shading tunnel after grafting. Lighting represents tomato seedlings recovered in an artificial illumination cultivation incubator after grafting. Data are the means of at least three biological replications. Different letters indicate a significant difference (LSD) test (α = 0.05). (**B**): After 6 d of healing and acclimatization using shading (top pictures) and lighting (bottom pictures) methods.

Using different recovery methods, grafted tomato seedlings showed different growth characteristics (Figure 1A,B). Compared with the traditional shading method, the plant height, petiole length of the first leaf, distance of the cut to the first leaf, and internode length of the grafted seedlings under artificial light treatment significantly decreased after 6 d and 8 d of recovery (Figure 1A). However, the shoot dry weight and healthy index of tomato seedlings treated with artificial lighting were significantly increased by 78.5% and 34.4% and 150.5% and 122.8% after 6 d and 8 d of grafting recovery, respectively (Figure 1A). For the stem’s diameter and shoot’s fresh weight, there were no significant differences between using artificial light and traditional shading methods (Figure 1A).

### 2.2. Influence of Different Recovery Methods on Grafted Tomato Seedling Leaf Gas Exchange Parameters and Chlorophyll Fluorescence Parameters 

**Figure 2:**
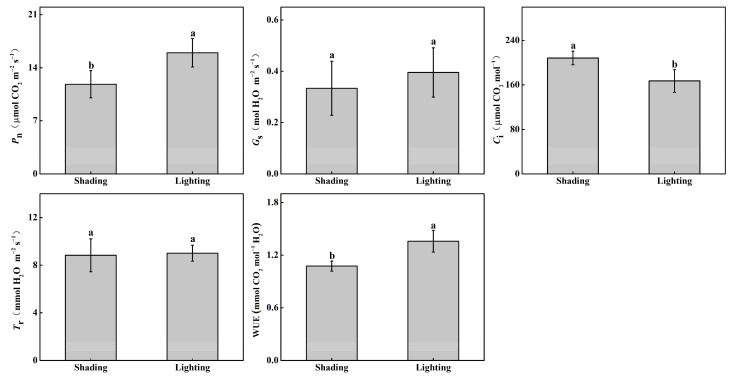
Effect of different recovery methods on net photosynthetic rate (P_n_), stomatal conductance (G_s_), intercellular CO_2_ concentration (C_i_), transpiration rate (T_r_), and water use efficiency (WUE). Shading represents tomato seedlings recovered in a shading tunnel after grafting. Lighting represents tomato seedlings recovered in an artificial illumination cultivation incubator after grafting. Data are the means of at least three biological replications. Different letters indicate a significant difference (LSD) test (α = 0.05).

The leaf net photosynthetic rate (P_n_) and water use efficiency (WUE) of tomato seedlings that were recovered in the artificial illumination cultivation incubator significantly increased by 35.0% and 26.4%, respectively, compared with seedlings obtained via traditional recovery methods using shading and moisturizers (Figure 2). The reverse results were revealed, with a significant reduction of 19.8% in intercellular CO_2_ concentration (C_i_) compared with the shading treatment. However, no significant difference was observed with respect to the stomatal conductance (G_s_) and transpiration rate (T_r_) between the two treatments.

**Figure 3:**
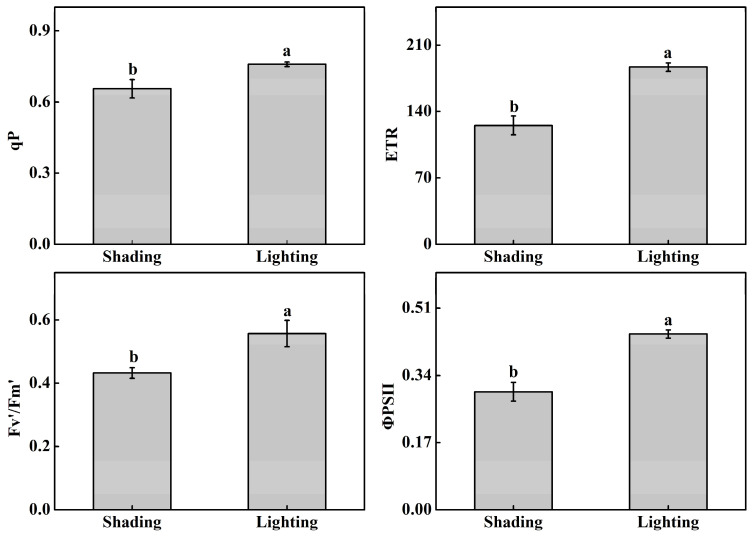
Effect of different recovery methods on the photochemical quenching coefficient (qP), electron transport rate (ETR), effective quantum yield of PSII photochemistry (Fv’/Fm’), and actual photochemical efficiency of PSII (ΦPSII). Shading represents tomato seedlings recovered in a shading tunnel after grafting. Lighting represents tomato seedlings recovered in an artificial illumination cultivation incubator after grafting. Data are the means of at least three biological replications. Different letters indicate a significant difference (LSD) test (α = 0.05).

The photochemical quenching coefficient (qP), electron transport rate (ETR), effective quantum yield of PSII photochemistry (Fv′/Fm′), and actual photochemical efficiency of PSII (ΦPSII) significantly increased by 15.6%, 49.0%, 28.6%, and 49.0%, respectively, when comparing lighting with shading treatments (Figure 3).

### 2.3. Influence of Different Recovery Methods on Grafted Tomato Seedling Chlorophyll and Carotenoid Contents

**Figure 4:**
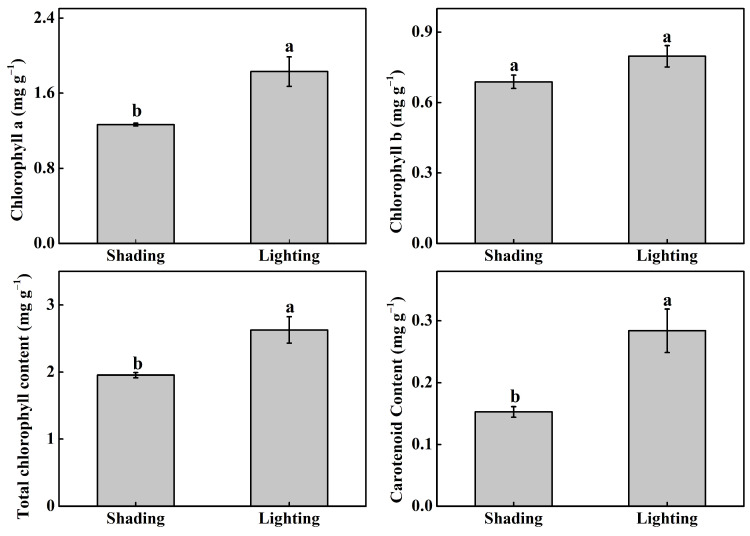
Effect of different recovery methods on chlorophyll a content, chlorophyll b content, the total chlorophyll content, and carotenoid content. Shading represents tomato seedlings recovered in a shading tunnel after grafting. Lighting represents tomato seedlings recovered in an artificial illumination cultivation incubator after grafting. Data are the means of at least three biological replications. Different letters indicate a significant difference (LSD) test (α = 0.05).

Chlorophyll a (Chl), Chl b, total Chl, and carotenoid contents significantly increased by 44.7%, 15.8%, 34.5%, and 86.0%, respectively, when comparing lighting with shading treatments (Figure 4).

### 2.4. Transcriptome Sequencing and Assembly 

Transcriptome sequencing was performed on the lighting- and shading-treated leaves using the Illumina high-throughput platform. Subsequently, 6.4 × 10^7^ and 5.9 × 10^7^ raw reads were obtained, and 6.2 × 10^7^, and 5.7 × 10^7^ clean reads were obtained after screening, respectively (Table 1). The overall data sequencing error rate was 0.03% for all leaves. Additionally, for shading- and lighting-treated leaves, the Q30 was 92.0% and 91.9%, and the GC content was 44.4% and 43.8%, respectively. The total mapped rate and uniquely mapped rate for the lighting- and shading-treated leaves were 97.3% and 97.5% and 93.2% and 93.5%, respectively (Table 1).

### 2.5. Gene Ontology (GO) Enrichment Analysis of DEGs

**Figure 5:**
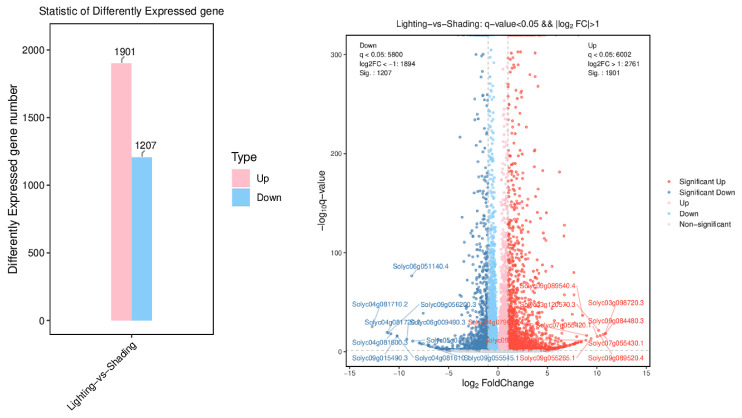
Number of DEGs detected between the shading and lighting seedlings. Shading represents tomato seedlings recovered in a shading tunnel after grafting. Lighting represents tomato seedlings recovered in an artificial illumination cultivation incubator after grafting.

**Figure 6:**
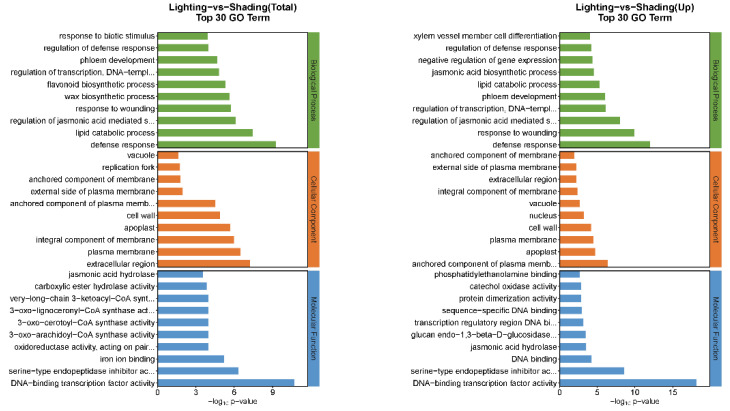
GO statistics and significant enrichment analysis of the identified DEGs detected between the shading and lighting seedlings. Shading represents tomato seedlings recovered in a shading tunnel after grafting. Lighting represents tomato seedlings recovered in an artificial illumination cultivation incubator after grafting.

In total, 3108 DEGs with 1901 upregulated and 1207 downregulated genes were identified for lighting vs. shading (Figure 5). The GO function annotation of DEGs was carried out based on three categories: biological process, cellular component, and molecular function (total top 30). Among them, the upregulated genes for lighting treatment were significantly enriched in three major categories compared with the shading treatment (Figure 6). For the biological process, the total and upregulated genes for the lighting treatment were both most abundant in defense response compared with the shading treatment. The most enriched in the cellular component were the extracellular region genes, and the upregulated genes were enriched in the anchored component of the plasma membrane and apoplast. For molecular functions, they were mainly enriched in DNA binding transcription factor activity genes, and genes such as DNA binding transcription factor activity and serine-type endopeptidase inhibitor activity were also significantly upregulated.

### 2.6. Kyoto Encyclopedia of Genes and Genomes (KEGG) Pathway Enrichment Analysis of DEGs

**Figure 7:**
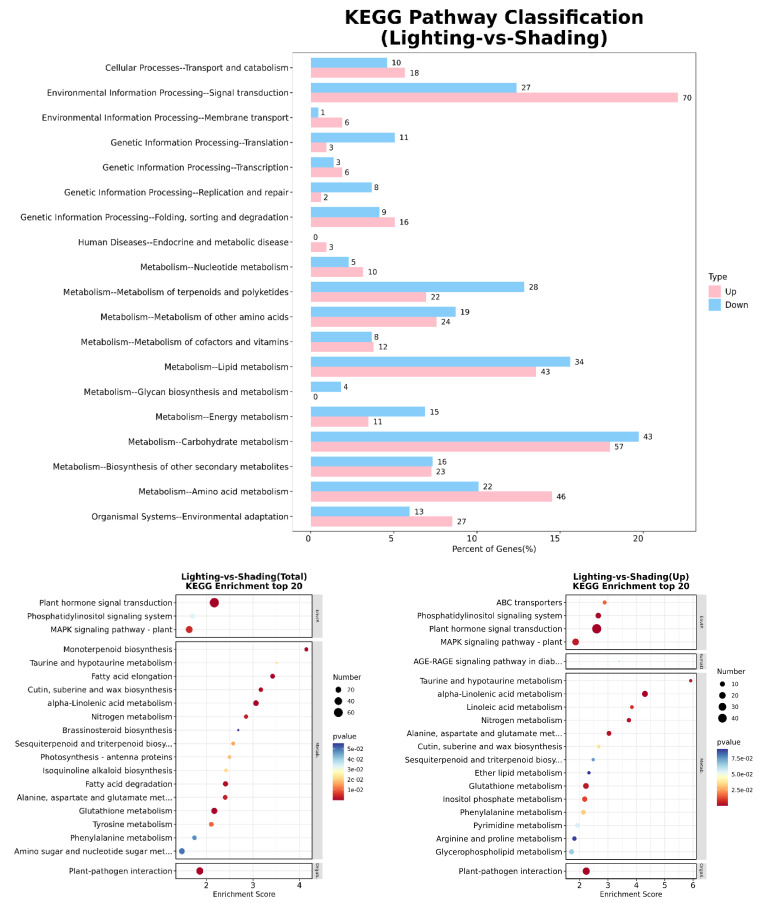
Significantly enriched KEGG pathways among the DEGs detected between shading and lighting seedlings. Shading represents tomato seedlings recovered in a shading tunnel after grafting. Lighting represents tomato seedlings recovered in an artificial illumination cultivation incubator after grafting.

The top 20 pathway enrichment DEGs annotated using the KEGG pathway database when comparing the lighting and shading treatments are shown in Figure 7. The upregulated DEGs were mainly associated with plant hormone signal transduction (70) and carbohydrate metabolism (57), as well as amino acid metabolism (46), lipid metabolism (43), and environmental adaptation (27). On the other hand, the enriched downregulated DEGs were intensively involved in carbohydrate metabolism (43), lipid metabolism (34), metabolism of terpenoids and polyketides (28), plant hormone signal transduction (27), and amino acid metabolism (22).

### 2.7. Effect of Different Recovery Methods on the Expression of Plant-Hormone-Signaling-Related Genes

**Figure 8:**
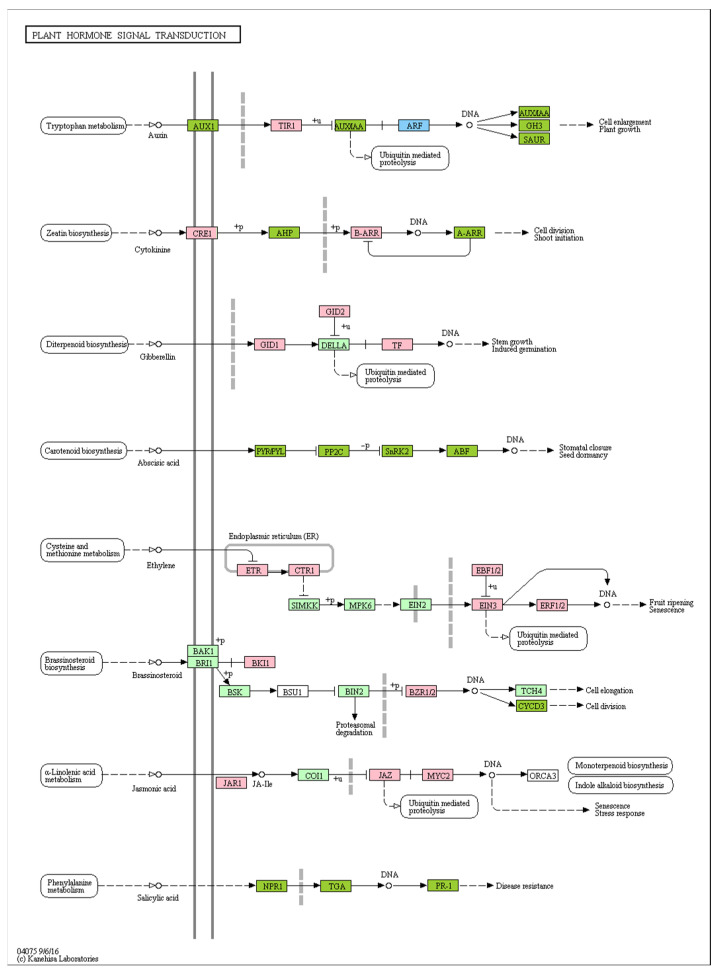
Upregulated genes in the plant hormone signal transduction pathway (shading vs. lighting). Upregulated genes are indicated by the red boxes. Shading represents tomato seedlings recovered in a shading tunnel after grafting. Lighting represents tomato seedlings recovered in an artificial illumination cultivation incubator after grafting.

Plant hormone signal transduction is dramatically affected by different grafting recovery methods. The KEGG pathway analysis indicated that 70 and 27 plant hormone signal transduction genes had higher and lower expression levels, respectively, for the lighting-treated leaves than for shading-treated leaves. The DEGs were mainly associated with the defense response, response to wounding, jasmonic-acid-mediated signaling pathway, regulation of defense response, auxin response, cytokinin response, cell division, growth regulation, gibberellin response, etc. (Figure 8). Plant hormone signal transduction genes related to auxin regulated cell enlargement, cytokinin regulated cell division, gibberellin regulated stem growth, abscisic acid regulated stomatal closure, and jasmonic acid and salicylic acid regulated stress response and resistance (Figure 8).

### 2.8. Expression Analysis of Homologous Lighting-Responsive Genes

**Figure 9:**
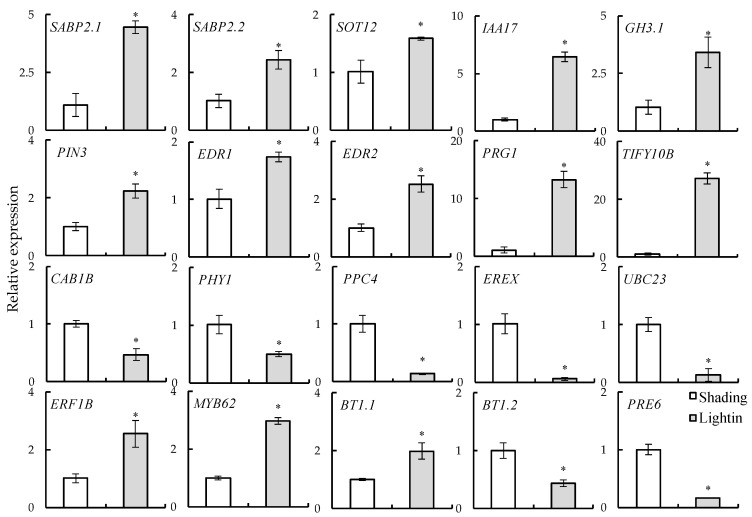
Real-time fluorescence quantitative PCR analysis of the gene expression patterns in grafted tomato seedlings under shading and lighting treatments. Shading represents tomato seedlings recovered in a shading tunnel after grafting. Lighting represents tomato seedlings recovered in an artificial illumination cultivation incubator after grafting. Values are expressed as the mean ± SD (*n* = 3). The difference between the control and treatment groups was statistically significant (* *p* < 0.05).

To evaluate the expression patterns of homologous genes in encoding light response, we searched 20 light-responsive genes in the tomato transcriptome database. Using the homologous ToEIF4 gene as an internal control, we analyzed the expression patterns of 20 light-responsive genes in tomato leaves via qRT-PCR (Figure 9). The results show that 13 light-responsive genes were significantly upregulated, and 7 exhibited significant downregulation under light conditions, which was similar to the consistent trends of their transcriptome. The correlation analysis results indicate that the response of grafted tomato seedling leaves to light involves multiple regulatory pathways, including transcription, photosynthesis, hormones, and signals.

### 2.9. Effect of Different Recovery Methods on Plant Hormone Content

Salicylic acid (SA) and the kinetin contents (Kin) of lighting-treated seedlings increased significantly by 140.9% and 116.8%, respectively, compared with shading-treated seedlings, while indoleacetic acid (IAA) and the jasmonic acid contents (JA) of lighting-treated seedlings decreased significantly by 33.8% and 48.7%, respectively, compared with shading-treated seedlings. There were no significant differences between aminocyclopropanecarboxylic acid (ACC), trans-zeatin (tZ), and abscisic acid contents (ABA) in the two treatments (Table 2).

## 3. Discussion

### 3.1. Light Can Enhance the Healthy Index of Grafted Tomato Seedlings under Recovery

The results of our study showed that lighting significantly increased the tomato seedling shoot dry weight and healthy index of tomato seedlings and decreased the plant height, petiole length of the first leaf, distance of the cut to the first leaf, internode length, and shoot fresh weight (Figure 1A,B). Healthy indexes can quantitatively and objectively evaluate the quality of seedlings [15]. Seedlings under the lighting treatment exhibited substantially higher healthy indexes. This suggests that these seedlings are better suited for future high production in cultivation greenhouses, as supported by Liu et al. [16], who found that seedlings with higher seedling indexes are more conducive to cultivating high-quality tomatoes. The tomato is a photophilous vegetable, and it is sensitive to low-light stress; the optimum irradiance of tomatoes is 500–800 μmol m^−2^ s^−1^, and the optimal light intensity requirement for growing tomatoes indoors is 400–500 μmol m^−2^ s^−1^ [17]. The results align with the research indicating that low light resulted in plant spindling, the low accumulation of biomass, and weak seedlings [18,19], which could significantly reduce the yield of tomatoes [20].

### 3.2. Light Can Improve the Photosynthesis and Chlorophyll Fluorescence Parameters of Grafted Tomato Seedlings under Recovery

Light intensity is a major environmental factor in photosynthesis and plant growth. The health of a plant and its normal physiological characteristics are reflected by high levels of photosynthesis [21]. In our study, we found that the P_n_ and WUE of grafted tomato seedlings that recovered under lighting treatment significantly increased compared with shading treatment seedlings (Figure 2). This is mainly due to light directly affecting plant photosynthesis and photomorphogenesis. Long-time low-light stress decreases photosynthesis in leaves by disrupting photosynthetic organelles. Khan et al. [20] also revealed that by increasing the production of light-harvesting complex II (LHCII), more light energy was absorbed for photosynthesis to better adapt to the low-light stress. 

Compared with the shading treatment, the qP, ETR, Fv′/Fm′, and ΦPSII of the lighting treatment significantly increased during the healing and acclimatization period. Chlorophyll fluorescence has developed into a well-established, non-invasive technique for studying photosynthesis signals and the injury to the photosystem. The measured parameters in this study elucidated that tomato seedlings were exposed to stress to some extent under shading treatment [22,23]. Plants grown in low-light environments exhibit lower levels of photosystem (PS) II, ATP synthase, cytochrome (Cyt) b/f, and ribulose-1,5-bisphosphate carboxylase/oxygenase (Rubisco), as well as reduced electron transport and CO_2_ consumption [24].

Chl a is essential for photochemistry, while Chl b provides plants an advantage in harvesting light around 450 nm, a wave region of light that is not efficiently absorbed by Chl a [20]. Carotenoids can transfer light energy to chlorophyll-a for converting to light energy and protecting photosynthetic plant systems [25,26]. Chl a, Chl b, total Chl, and carotenoid contents significantly increased under lighting treatments. Chl is a major pigment used by plants for capturing light energy, and it is the most important pigment in photosynthesis and most responsive to different environmental stresses [27]. The results of the study show why shading treatment resulted in low photosynthesis and biomass.

### 3.3. Light Affects Many Signal Pathways of Grafted Tomato Seedlings under Recovery

It has been observed that grafting can lead to alterations in the transcriptome profile of parent plants [28,29]. However, the focus has primarily been on the relationship between a graft union and the enhancement of stress tolerance in various plants [30,31]. To further explore the underlying mechanism of lighting-promoted recovery after grafting, the transcriptomes of lighting and shading seedlings were sequenced using the Illumina Novaseq 6000 sequencing platform. During the comparison between lighting and shading treatments, a total of 3108 DEGs were identified, consisting of 1901 upregulated and 1207 downregulated genes. The DEGs underwent functional characterization based on the enriched GO terms. The upregulated genes under the lighting/shading treatments were found to be enriched in functions such as defense response, anchored component of the plasma membrane, and DNA binding transcription factor activity. The increased expression of related genes may be attributed to the reconstruction of the cell wall, polysaccharide binding, protein folding, cell reorganization, and the transfer of substances from the rootstock to the scion [32].

The enriched KEGG pathways indicated that the expression levels of certain plant hormone signal transduction genes were significantly upregulated under lighting compared with the corresponding expression under shading treatment. These genes were mainly related to auxin, which regulated cell enlargement; cytokinin, which regulated cell division; gibberellin, which regulated stem growth; abscisic acid, which regulated stomatal closure; and jasmonic acid and salicylic acid, which regulated stress response and resistance. This was similar to the results of KEGG pathway enrichment analyses, which indicated that the plant hormone signal transduction genes were significantly enriched following the transcriptome sequencing of grafted tomato by Wang et al. [33], along with the hormonal balance relating to plant hormone signal transduction genes, which was revealed by the transcriptome sequencing of grafted citrus performed by He et al. [34]. Plant hormones play an important role not only in the grafting healing process and vascular formation process but also in maintaining growth between the scion and the stock [31,35].

In the transcriptome data of grafted tomato seedling recovery after light treatment, we selected 20 genes for qRT-PCR verification (Figure 9). Among them, 10 genes involved in the plant hormone signaling pathway were upregulated and contained salicylic acid-binding protein 2 (SABP2), cytosolic sulfotransferase 12 (SOT12), probable indole-3-acetic acid-amido synthetase (GH3.1), auxin-responsive protein (IAA17), auxin efflux carrier component 3 (PIN3), serine/threonine–protein kinase (EDR1), protein ENHANCED DISEASE RESISTANCE 2 (EDR2), Pto-responsive gene 1 protein (PRG1), and protein TIFY 10b. SABP2.1/2.2 and SOT12 are involved in the salicylic acid metabolic pathway, and SABP2 can convert methyl salicylate (MeSA) into SA as part of the signal transduction pathway that activates systemic acquired resistance within system tissues [36]. GH3.1, IAA17, and PIN3 participate in the auxin metabolic pathway, and GH3.1 can catalyze the synthesis of indium-3-acetic acid (IAA) amino acid conjugate in response to the excess auxin mechanism in plants [37]. IAA17 acts as an inhibitory transcription factor of early auxin response genes at low auxin concentrations, and it interacts with ARFs to form heterodimers that may change their ability to regulate the expression of early auxin response genes [38]. As a component of the auxin effector vector, PIN3 participates in the lateral auxin transport system and mediates plant growth [39]. There are also potential mechanisms of interaction between auxin and light reaction pathways. The increased expression levels of EDR1 and EDR2 also indicated that ABA or ET signaling pathways were positively regulated during tomato grafting, and PRG1 and TIFY10B were also actively involved in the JA pathway. These results were similar to those of KEGG analysis transcriptome results. Transcriptome data showed that the metabolic pathways involved in photosynthesis were inhibited, and we verified that the expression levels of three genes were significantly downregulated, including the a–b binding protein 1B (CAB1B), light-sensor protein kinase (PHY1), and phosphoenolpyruvate carboxylase 4 (PPC4). This indicates that CAB1B, as a photoreceptor [40], captures and transmits excitation energy to the photosystem during the recovery of tomato grafting, but mechanical damage still causes certain damage to the photosynthetic system of seedlings. Among the transcriptome data, most transcription factors were upregulated for the light restoration of tomato grafting. We further verified five transcription factors. Among them, transcription factor MYB62, ethylene responsive transcription factor 1B (ERF1B), and BTB/POZ and TAZ domain-containing protein 1.1 (BT1.1) gene expression levels increased. The expression levels of transcription factor PRE6 and BT1.2 were decreased. In the course of plant evolution, plants have acquired a variety of abilities to respond to or resist stress, among which transcription factors play an important role in regulating abiotic stress tolerance. For example, MYB transcription factors, as one of the largest transcription factor families with complex functional differentiation in plants, selectively expand in plants, and the members of the MYB family play roles in a variety of plant-specific processes [41]. They can also form regulatory complexes with bHLH transcription factors. It is a key factor in regulatory networks that controls development, metabolism, and responses to biotic and abiotic stresses.

### 3.4. Light Influences the Hormone Level of Grafted Tomato Seedling under Recovery

Phytohormones regulate every aspect of plant development and response to biotic and abiotic stresses. Plant hormones function as the governors of signal events to create a complex network of hormonal interactions [35,42]. In the current study, we observed a significant increase in the salicylic acid (SA) and kinetin contents (Kin) of lighting seedlings, in accordance with the KEGG pathway enrichment analyses results. SA is among the wide variety of phenolic compounds bearing a hydroxyl group or its derivative, which is synthesized by plants, and it is an important plant hormone that regulates many aspects of plant growth and development, such as being involved in cell growth, seed germination, seedling establishment, stomatal closure, respiration, senescence-associated gene expression, nodulation in legumes, and fruit yield, as well as resistance to abiotic or biotic stress [43,44,45]. SA can alter the synthesis of and/or signaling by other plant hormones, including jasmonic acid (JA), ethylene (ET), and auxin [44]. Hence, we believe that the significant increase in SA is one of the main reasons why lighting tomato seedlings had better survival rates and quality during the healing and acclimatization stages after grafting [46]. Padilla et al. [47] found that a drastic SA increase occurred immediately after water stress was applied to grafted peppers, which is associated with changes in the hormonal balance. Fu et al. [48] revealed that SA is involved in rootstock–scion communication and that grafting can induce chilling tolerance in cucumber plants under chilling stress. Kin is an endogenous cytokinin (CK) that not only promotes cell division but also delays the senescence of detached leaves, induces bud differentiation and development, and increases stomatal opening. The high Kin content of lighting tomato seedlings had high biomass and better growth, and this is related to the CKs that regulate plant growth and development by modulating key physiological and molecular processes, especially the development of the vascular system [49,50,51]. Ikeuchi et al. [52] concluded that CK biosynthesis genes were upregulated, CK levels increased, and CK response was enhanced during wounding in Arabidopsis. An exogenous application of CKs has been reported to stimulate the formation of vascular strands at the graft union site, thereby enhancing vascular reconnection during grafting [35]. Kin application has also been shown to decrease the toxic effects by interacting with salicylic acid, gibberellic acid, jasmonic acid, and abscisic acid [53], promoting the restoration of primary and secondary metabolite status and the upregulation of antioxidant defense systems, thus alleviating the harmful effects of UVC stress [54].

Our results revealed that both the IAA and JA of lighting-treated seedlings decreased significantly compared with the shading treatment. Furthermore, the corresponding measurements of plant height, the petiole length of the first leaf, distance of the cut relative to the first leaf, internode length, and shoot fresh weight of the lighting treatment decreased significantly. Wang et al. [55] also found that a long period of shading resulted in a rapid increase in IAA content in order to promote stem elongation mainly because of the characteristics of IAA. Shading entails a stronger reduction in red and blue than far-red and green wavelengths [56]; meanwhile, it decreases the activity of phytochrome B and cryptochrome 1, resulting in the initiation of shade avoidance responses, such as the promotion of stem and petiole elongation [57,58]. Similar results were found by Han et al. [59], where severe shading produced significantly increased leaf IAA content in oilseed peonies. JA accumulated in response to wounding [60]. A cross-talk between JA and IAA signal pathways was found in the regulation of cell division during tissue reunion, but little is still known about the role of JAs during grafting [35]. We show that the high content IAA and JA of seedlings is mainly because of slow healing and low-light intensity under shading treatments. Our results show that there were no significant differences in ACC, tZ, and ABA between shading and lighting treatments, which may be because the plants had been acclimated in a normal greenhouse for a couple of days.

## 4. Materials and Methods

### 4.1. Plant Material and Treatments

The commercial tomato hybrid *Solanum lycopersicum* cv. Saopolo was used as the scion, and Maxifort was evaluated as the rootstock. The seeds of Saopolo and Maxifort were sowed directly in rockwool plugs (cylinder diameter = 20 mm; height = 27 mm) and then covered with vermiculite substrate and germinated at 25 °C in the germination chamber (22 August 2022). We made sure that the plugs in the trays (240 plugholes) were well watered with 1.5–2.0 dS m^−1^ EC-level water. The pH level of the water needed to be 5.5–6.0. The relative humidity of all germination chambers was 100%. The trays were taken to the seedling greenhouse (day temperature maintained at 25–30 °C via a high-pressure fog and air conditioner; night temperature was kept at 23–25 °C via the air conditioner) until 50% of the plants were visible (this took approximately 2–3 days). The seedlings were grafted when the scion and stock seedlings had both developed 2 true leaves and a stem diameter of about 2 mm after 15 days of sowing. The rootstocks of Maxifort half-seedlings were transplanted to another tray evenly, and the 120 plugs were maintained in one tray for at least 2 days before grafting, ensuring they were easy to graft.

**Figure 10:**
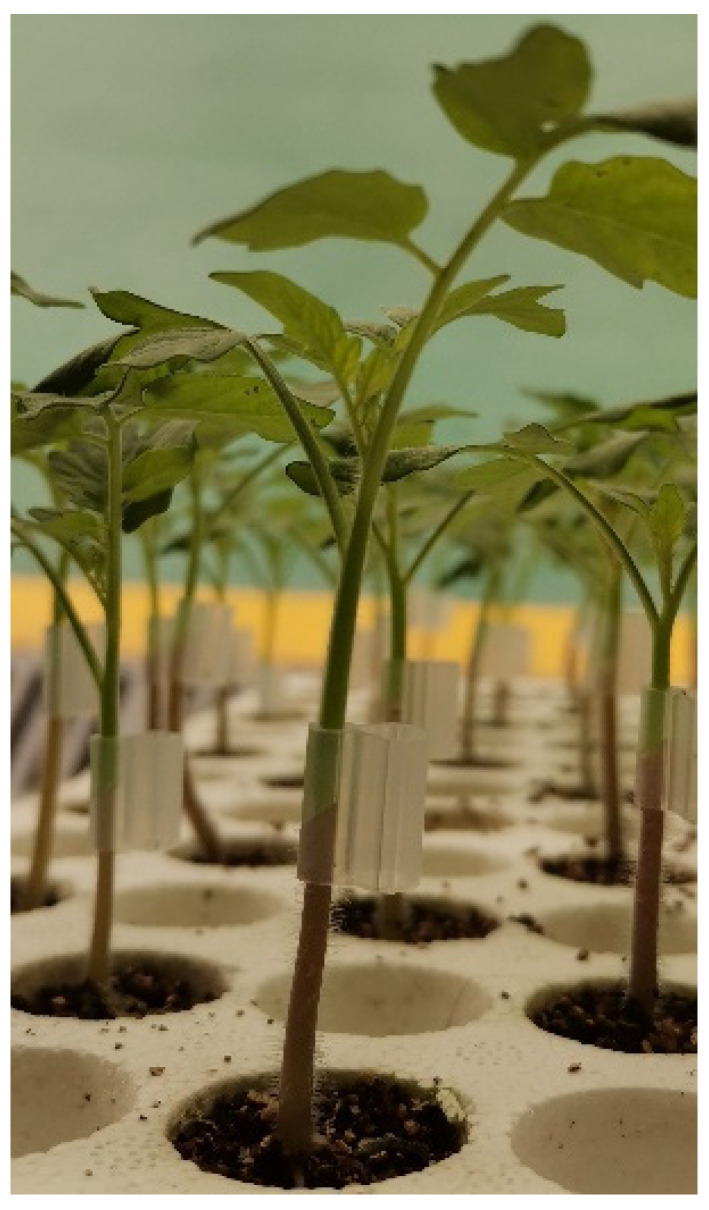
Size of the grafted seedlings and grafting method.

Grafting was performed using the splicing method. The rootstock seedlings were cut about 1 cm below the cotyledon leaves, and scion seedlings were cut about 0.5 cm above the cotyledonary leaves. The scions and rootstocks were cut at an approximate angle of 45° (Figure 10). Grafts were made immediately after cutting the plants, and grafting clips were used to adhere to the graft union [61].

**Figure 11:**
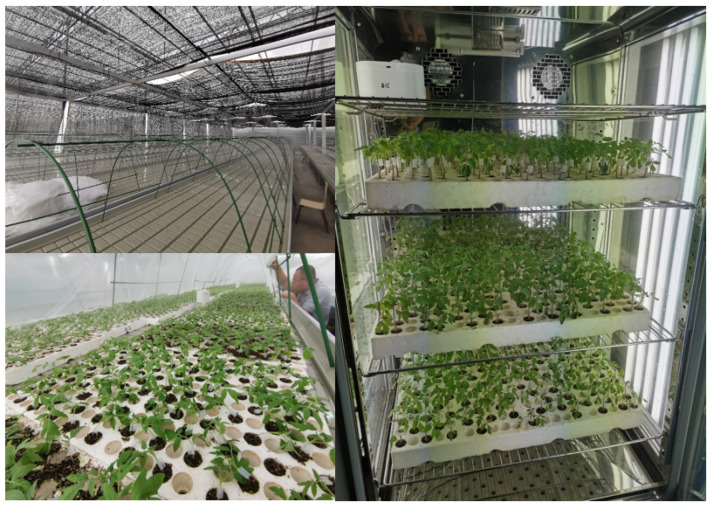
Grafted tomato seedlings at the healing and acclimatization stages for the shading (left pictures) and lighting (right pictures) treatments. Shading represents tomato seedlings recovered in a shading tunnel after grafting. Lighting represents tomato seedlings recovered in an artificial illumination cultivation incubator after grafting.

The experiment comprised two treatments (Figure 11): (1) shading treatment: traditional recovery with shading and moisturizer. The grafted tomato seedlings were recovered in the little tunnel with plastic films and moisturizers, and a larger shading curtain (80% shading) was used for coverage above the little tunnel. The grafted seedlings were maintained at about 23–28 °C by a high-pressure fog and air conditioner and relative humidity (RH) of 100% in the little tunnel for 3 days after grafting. Then, the plastic films for ventilation were gradually opened, and RH was kept at 90–100%. All grafted seedlings were transplanted to the seedling greenhouse (normal condition) after 6 days of grafting. (2) Lighting treatment: recovery in the artificial illumination cultivation incubator (small plant factory). Grafted tomato seedlings were grown in the artificial illumination cultivation incubator with no light on the first day (15–16 h) of recovery, and the temperature and relative humidity were set at 25 °C and 100%, respectively. In total, 100 µmol photon m^−2^ s^−1^ was delivered on the second recovery day (24 h), and the temperature was set at 25 °C during the day (3 h) and 23 °C during the night (3 h). Then, 100 µmol photon m^−2^ s^−1^ was delivered on the third (24 h) recovery day, and the temperature was set at 25 °C during the day (5 h) and 23 °C during the night (5 h). Moreover, 200 µmol photon m^−2^ s^−1^ was delivered on the fourth (24 h) recovery day, and the temperature was set at 25 °C during the day (5 h) and 23 °C during the night (5 h). Then, 300 µmol photon m^−2^ s^−1^ was delivered on the fifth (24 h) recovery day, and the temperature was set at 25 °C during the day (5 h) and 23 °C during the night (5 h). Finally, 300 µmol photon m^−2^ s^−1^ was delivered on the sixth recovery day, and the temperature was set at 25 °C during the day (10 h) and 23 °C during the night (5 h). Then, the grafted seedlings were transplanted to the seedling greenhouse (normal conditions). The RH was set at 100% at 1–3 days and 90–100% at 3–6 days, and a moisturizer was used to maintain the high relative humidity. The largest leaves were collected 8 days after grafting for each treatment, and the samples were immediately frozen in liquid nitrogen for further analysis.

### 4.2. Growth Parameter Measurement

The plant height, stem diameter, petiole length of the first leaf, distance of the cut to the first leaf, and internode length were measured after 6 and 8 days of treatments. At the same time, the shoots of the plants under each treatment were collected, their fresh weight was measured, and then they were placed in an oven at 80 °C for 3 days to measure the corresponding dry weight. The health indexes of the seedling were calculated according to Zhou et al. [15] and modified using the following equations: Healthy index of seedling = [(Stem diameter/Plant height) × Dry mass of shoot].

### 4.3. Measurement of Chlorophyll and Carotenoid Contents

Of the leaf tissue, 0.1 g was taken from the leaves under each treatment and soaked in 10 mL of 95% ethanol until the leaves faded to white. Then, a UV-visible spectrophotometer (Ultraviolet-2700; Shimadzu, Tokyo, Japan) was employed to measure the Chl a, Chl b, and Car contents at 665, 649, and 470 nm, respectively, as calculated by Jiang et al. [62] with modifications. Three biological replicates were performed for each treatment.

### 4.4. Leaf Gas Exchange and Chlorophyll Fluorescence Analysis

The portable photosynthesis system (CIRAS-3, PP Systems, Amesbury, MA, USA) equipped with a leaf chamber fluorometer (PLC3 Universal Leaf Cuvette, 18 × 25 mm window, CFM-3) was used to measure the gas exchange parameters and chlorophyll fluorescence of leaves, including the net P_n_, G_s_, C_i_, T_r_, and qP, ETR, Fv′/Fm′, and ΦPSII. The irradiance level was set at 1000 µmol photon m^−2^s^−1^. The air temperature, CO_2_ concentration, and relative humidity were set according to greenhouse conditions. The leaves were acclimated to the irradiance level for approximately 1–2 min prior to the measurements. The measurements were conducted between 9 and 11 a.m., 8 days after transplanting. Chlorophyll fluorescence measurements were performed using the same leaves as the gas exchange measurements, following the description by Jiang et al. [63]. Each leaf was measured twice for photosynthesis and fluorescence parameters, and at least six leaves were measured for each treatment.

### 4.5. RNA Extraction and Library Preparation

Total RNA was extracted from the treatment group using the TRIzol reagent (Invitrogen, Carlsbad, CA, USA). RNA purity and quantification were assessed using the NanoDrop 2000 spectrophotometer (Thermo Scientific, Waltham, MA, USA). RNA integrity was evaluated using the Agilent 2100 Bioanalyzer (Agilent Technologies, Santa Clara, CA, USA). The libraries were constructed according to the instructions of the VAHTS Universal V6 RNA-seq Library Prep Kit. Transcriptome sequencing and analysis were performed by OE Biotech Co., Ltd. (Shanghai, China).

### 4.6. Bioinformatic Analysis

The libraries were subjected to sequencing using the Illumina Novaseq 6000 platform. Subsequently, raw data (raw reads) were processed using fastp software to remove low-quality reads and obtain clean reads [64]. The clean reads were then mapped to a reference genome using HISAT2 [65]. The fragments per kilobase of transcript per million mapped read (FPKM) values of each gene were calculated using cufflinks [66], and the counts of reads for each gene were obtained via htseq-count [67]. Differential expression analysis was performed using DESeq 2 [68], with q-value < 0.05 and fold change > 2 or <0.5 used as the threshold for significant DEGs. A hierarchical cluster analysis of DEGs was conducted to examine the expression pattern of genes. GO enrichment and KEGG pathway enrichment analysis of DEGs were performed using R (v 3.2.0) based on the hypergeometric distribution [62].

### 4.7. Hormone Detection Assays

The quantitative detection of hormones, SA, Kin, IAA, JA, ACC, tZ, and ABA was in accordance with the previously published paper, with modifications [69], and this was conducted via ultra-performance liquid chromatography–electrospray ionization tandem mass spectrometry (UPLC–ESI–MS/MS) at Shanghai Luming Biotechnology Co., Ltd. (China). Of the samples, 50 mg was taken from each treatment group, ground into powder, and a mixture buffer of 2-propanol/H_2_O/concentrated HCl (2:1:0.002, vol/vol/vol) was used as the extraction solvent. The sample solution was placed in a reverse-phase C18 Gemini HPLC column for HPLC–ESI–MS/MS (AB SCIEX, Framingham, MA, USA) analysis. The conditions and setup of the HPLC–ESI–MS/MS and MRM were performed as described previously [62,70].

### 4.8. Statistical Analysis

Statistical analysis was conducted using SAS version 9.3 software (SAS Institute Inc., Cary, NC, USA). Each value is represented as the mean ± standard deviation, with at least three replicates. The differences between treatment methods were tested using the least significant difference (LSD) method at a significance level of α = 0.05. The data were plotted using Origin 8.5 software (Origin Lab, Northampton, MA, USA).

## 5. Conclusions

As a widely used technique to enhance the phenotype of tomatoes, grafting can effectively control soil-borne diseases and alleviate biotic and abiotic stress factors within practical production. Traditionally, tomato seedlings are grafted and adapted to a shaded environment. On the other hand, the evolutionary mechanisms underlying grafting remain mysterious. In this study, our results confirm that artificial light played an important role in promoting the healing and adaptation of grafted seedlings during the early stage. Compared with grafted tomato seedlings adapted under shading, those under artificial light had a high healthy index and exhibited higher contents of leaf chlorophyll, shoot dry weight, and P_n_ and WUE. Furthermore, transcriptome data showed that DEGs were mainly enriched in various metabolic pathways, such as plant hormone signaling, photosynthesis, transcription, protein folding and processing, protein synthesis, and protein degradation. The quantification of gene expression changes in transcriptome data via qRT-PCR showed similar results. GO analysis and KEGG pathway enrichment analysis showed that the plant hormone signal transduction pathway, as the most important metabolic process, affected the recovery of tomato seedlings grafted under lighting. Furthermore, the higher hormone content of SA and Kin was also one of the main reasons for the good growth of grafted tomato seedlings under artificial light. Therefore, artificial light is a meaningful environmental factor that promotes the rapid recovery and growth of grafted tomato seedlings.

## Figures and Tables

**Table 1:** Summary of the sequence reads for RNA samples, including the shading and lighting treatments.

	Raw Reads	Clean Reads	Error_Rate/%	Q30/%	GC_pct/%	Total Map/%	Unique Map/%
Shading	6.4 × 10^7^	6.2 × 10^7^	0.03	92.0	44.4	97.3	93.2
Lighting	5.9 × 10^7^	5.7 × 10^7^	0.03	91.9	43.8	97.5	93.5

**Table 2:** Effect of different recovery methods on plant hormone content.

Plant Hormone	Shading (ng/g)	Lighting (ng/g)
SA	830.91 ± 139.66 b	2001.44 ± 49.38 a
Kin	0.15 ± 0.003 b	0.326 ± 0.032 a
IAA	12.95 ± 2.06 a	8.57 ± 1.07 b
JA	1665.02 ± 252.21 a	853.59 ± 6.36 b
ACC	488.55 ± 45.14 a	470.29 ± 10.96 a
tZ	118.67 ± 15.26 a	106.21 ± 2.44 a
ABA	1179.33 ± 120.87 a	1385.07 ± 29.84 a

Data are the means of at least three biological replications. Different letters indicate a significant difference (LSD) test (α = 0.05).

## Data Availability

All data used during the study are available from the author Xiaotao Ding by request (e-mail: xiaotao198108@163.com).
